# Immunolocalization of TSOL18 and TSOL45-1A, the successful protective peptides against porcine cysticercosis, in *Taenia solium *oncospheres

**DOI:** 10.1186/1756-3305-4-3

**Published:** 2011-01-06

**Authors:** Joel Martinez-Ocaña, Mirza Romero-Valdovinos, Rina G de Kaminsky, Pablo Maravilla, Ana Flisser

**Affiliations:** 1 Hospital General "Dr. Manuel Gea Gonzalez", SSA. Calzada de Tlalpan 4800, Col Seccion XVI, Tlalpan, Mexico 14080 DF, Mexico; 2 Departamento de Pediatria, Facultad de Ciencias Medicas, Universidad Nacional Autonoma de Honduras, Tegucigalpa, Honduras; 3 Departamento de Microbiologia y Parasitologia, Facultad de Medicina, Universidad Nacional Autonoma de Mexico, Mexico 04510 DF, Mexico

## Abstract

*Taenia solium *life cycle includes humans as definitive hosts and pigs as intermediate hosts. One of the measures to stop the life cycle of this parasite is by vaccination of pigs. In experiments performed in pigs with TSOL18 and TSOL45-1A, two recombinant *T. solium *proteins, 99.5% and 97.0% protection was induced, respectively. The purpose of this paper was to localize these antigens in all stages of the parasite (adult worms, oncospheres and cysticerci) by immunofluorescence, with the use of antibodies against TSOL18 and TSOL45-1A that were obtained from the pigs used in the vaccination experiment. Results show that TSOL18 and TSOL45-1A are expressed on the surface of *T. solium *oncospheres and not in tapeworms or cysticerci, indicating that they are stage-specific antigens. This, therefore, might explain the high level of protection these antigens induce against pig cysticercosis.

## Findings

*Taenia solium *is a cestode parasite that causes human neurocysticercosis, a public health problem in developing countries. The life cycle includes the adult tapeworm that grows in the human small intestine and cysticerci, the larval stage, which lodge in pig skeletal muscle and brain. The tapeworm has a scolex that anchors in the intestinal mucosa and is followed by a long row of proglottids, the initial ones are smaller and immature, the middle ones are mature and contain sexual organs and the last segments are the biggest and are gravid because they contain around 60,000 eggs each one. Inside the egg a hexacanth embryo, called an oncosphere, is surrounded by an oncospheral membrane and an egg shell or embryophore; proglottids and eggs are liberated with faeces. After ingestion of eggs by swine, the intermediate host, oncospheres are released and liberated from their membrane, becoming activated in the gut in order to cross the intestinal mucosa and transform into cysticerci. When an individual eats insufficiently cooked infected pork meat, the tapeworm develops [1].

Humans can also acquire cysticercosis after ingesting eggs, this phenomenon is associated with poor health education and lack of sanitation; it is prevalent in pork eating countries of Latin America, Asia and Africa, generating neurocysticercosis, the most frequent and devastating parasitic disease of the brain [2]. Vaccines have been developed targeting the oncosphere and preventing establishment of the parasite in immunized pigs. The recombinant *T. solium *oncosphere proteins, designated as TSOL18 and TSOL45-1A, have been found to induce 99.5% and 97.0% protection respectively, in vaccine trials against the experimental challenge of pigs with *T. solium *eggs [3,4]. Thus, it is biologically relevant to identify the presence of these antigens in the developmental stages of *T. solium*.

In order to localize the antigens on the parasite, blood samples from the pigs that were vaccinated with TSOL18, TSOL45-1A or GST (glutathione S-transferase, as carrier protein), were obtained prior and after immunization [3]. Serum was separated and stored at -20ºC until use. Whole immunoglobulin G (IgG) was purified with a commercial kit (Montage Antibody Purification Prosep-A, Millipore, Bedford, MA, USA), the purified IgG was adjusted at 1 mg/ml. Adult *T. solium *worms were obtained in the experimental hamster model [5,6] and the scolex-neck, immature and mature proglottids were separated, included in Tissue-tek (Tissue Freezing Medium, Durham, NC, USA) and frozen at -70ºC. Cysticerci, obtained from a naturally infected pig, were similarly processed. Before use, 4 µm thick sections were obtained, placed on silane-treated slides, air dried for 30 min, fixed in methanol-acetone for 10 min and dried for 15 min at room temperature. Tissues were rehydrated with PBS, permeabilized with 0.2% Triton X-100 in PBS for 15 minutes, treated for antigen retrieval with pronase E for 10 min and blocked with PBS that contained 1% BSA with 10% goat serum for 1 h at room temperature. TSOL18 IgG, TSOL45-1A IgG, GST IgG or pre-immune IgG, diluted 1:100 in the same blocking solution was added to the slide until all the section was covered and incubated overnight at 4ºC. After washing with PBS-0.3%-Tween 20, sections were incubated with fluorescein isothiocyanate (FITC) labelled goat anti-pig IgG (Santa Cruz Biotechnology Inc, Santa Cruz, CA, USA) for 1 hour at 37ºC and, after washing, counterstained with propidium iodine (PI, Sigma, St. Louis, MO).

Gravid proglottids were recovered in different times from two human tapeworm carriers from Honduras, identified as *Taenia solium *by the number of uterine lateral branches and stored at 4ºC in PBS with 1% antibiotic and antimycotic solution for 15-30 days. For use, gravid segments were placed in a sieve, cut with fine sharp scissors and the fragments obtained were further teased to release eggs. The egg solution was centrifuged and washed in PBS several times. Embryophoric blocks were disrupted with 10% sodium hypochlorite by gently mixing them with a glass Pasteur pipette, when most oncospheres were released from the embryophore, they were washed with RPMI-1640 and were activated by incubation in artificial intestinal fluid (1% pancreatin, 0.2% anhydrous sodium bicarbonate and 1% pig bile in RPMI-1640) for 45 min at 37ºC. As described by Kyngdon *et al*. [7], activation was confirmed under the microscope because oncospheres exhibited active and rhythmic movements; they were further centrifuged (1,000 g, 10 min) and washed in fresh RPMI-1640, thrice. Fixation, permeabilization, antigen retrieval, incubation with antibodies and counterstaining was performed as described above but in Eppendorf tubes. After oncospheres were washed, centrifuged and gently mixed, three drops were placed per slide, allowing to air dry at room temperature. Tissue sections as well as embryos were covered with fluorescence mounting medium (Vectashield, Vector laboratories Inc. Burlingame, CA, USA) and a cover slide. Preparations were observed in a Nikon epifluorescence microscope.

**Figure 1:**
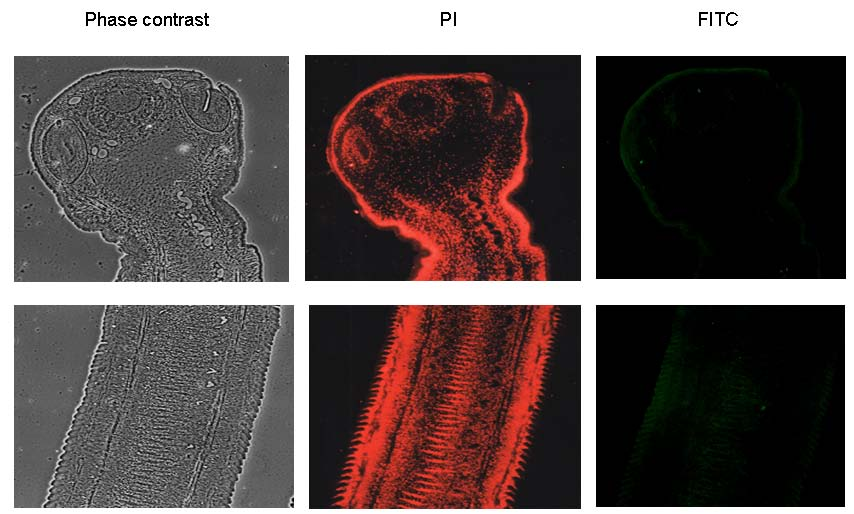
**Images of a scolex with the neck and several immature proglottids obtained from a *T. solium *tapeworm recovered from the experimental hamster model are seen under contrast phase microscopy or with filters for PI or FITC**. The first row corresponds to the section of the scolex-neck and the second row to proglottids. Only results with anti-TSOL18 are included because these, as those with anti-TSOL45-1A or GST, were negative. Magnifications 20×.

Figure 1 shows the results obtained with tapeworms recovered from the experimental hamster model seen under contrast phase microscopy or with filters for PI or for FITC. The first row corresponds to the section of the scolex-neck and the second row to proglottids. The worm can be clearly seen under phase microscopy, as well as with PI since the tissue was pre-fixed, but minimal reaction was found with FITC, indicating absence of specific staining. Since all samples had the same pattern, irrespectively of the IgG used (anti TSOL18, anti TSOL45, anti GST or pre-immune), only the samples incubated with TSOL18 are shown. Cysticerci were also negative (not shown).

**Figure 2:**
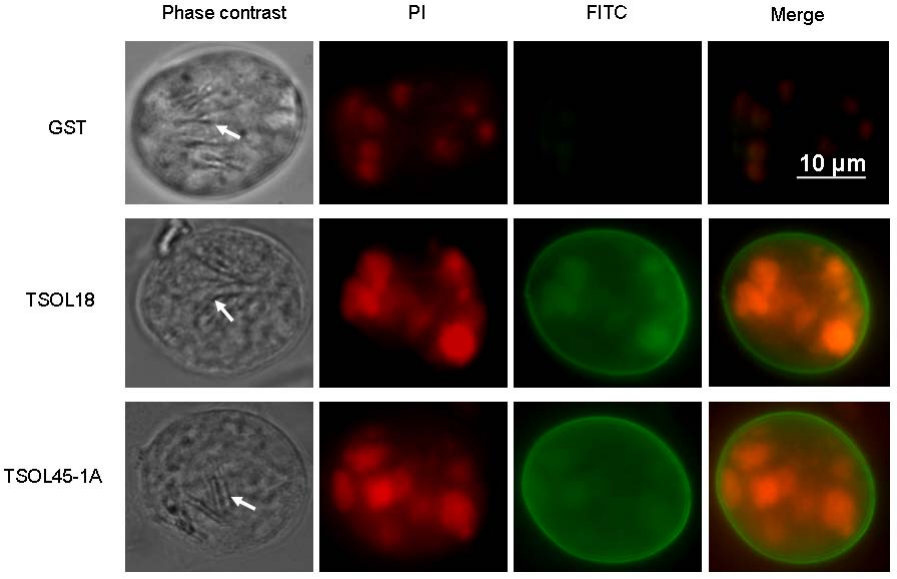
***Taenia solium *oncospheres obtained from a tapeworm carrier are seen under contrast phase microscopy, PI, FITC and a merged image of both stains**. The first row corresponds to oncospheres incubated with anti GST IgG as a negative control; rows 2 and 3 include images obtained with the specific anti TSOL18 and anti TSOL45-1A antibodies, respectively. The arrows in the first column point to the hooks of the oncospheres that was used for focus. Positive reactions were detected with anti-TSOL18 and anti-TSOL45-1A antibodies only on the surface of the oncospheres, as seen in the merged image. The high intensity emission of PI in FITC sections caused non-specific background fluorescence on the same internal structures seen in PI sections.

Positive reactions were only detected with anti-TSOL18 and TSOL45-1A IgG on the surface of oncospheres (Figure 2) with similar staining in different assays performed. In this case the first row corresponds to oncospheres incubated with anti GST IgG as a negative control; rows 2 and 3 include images obtained with anti TSOL18 and anti TSOL45-1A antibodies, respectively. The columns correspond to contrast phase microscopy, PI, FITC stains and merged images of both dyes. Pre-immune IgG was negative as was GST, therefore it is not shown. For the phase contrast figures, the hooks of the oncospheres (arrows) were set on focus, thus the rest of the organism is relatively out of focus. PI labelling was positive since, after activating the oncospheres, they were fixed as part of the staining process. Specific staining (FITC) was intensely detected only on the surface of the oncospheres.

Three protective antigens, To16, To18 and To45w, were found on the cytoplasm and the secretory granules of bilateral glandular cells of freshly hatched *T. ovis *oncospheres [8], this was further confirmed by quantitative immunogold labelling in penetration glands and secretory blebs [9]. A different staining pattern was found in activated oncospheres that were cultured for 7 days [8], which is similar to the one found in the present study, ie, on the surface of the oncospheres. This location agrees with the demonstration of *in vitro *capability of antibodies to destroy *T. solium *embryos [7]. Furthermore, although the precise time at which taeniid parasites from a challenge infection are killed by immune hosts is not known, *Taenia taeniaeformis *[10]*T. pisiformis *[11] and *Echinococcus granulosus *[12] oncospheres appear to be killed within 24 h after infection. These data indicate the need of exposed antigens in order to trigger an early protective immune response, supporting the importance of the presence of TSOL18 and TSOL45-1A on the surface of freshly hatched oncospheres.

## Abbreviations

TSOL18: the *T. solium *18 oncospheral protein; TSOL45-1A: the *T. solium *45-1A oncospheral protein; GST: Glutathione s- transferase; BSA: bovine serum albumin; To16: the *T. ovis *16 oncospheral protein; To18: *T. ovis *18 oncospheral protein; To45w: *T. ovis *To45w oncospheral protein.

## Competing interests

The authors declare that they have no competing interests.

## Authors' contributions

JMO and AF formulated the idea, MRV, PM and RGK participated during experimental process and in the discussion. All authors contributed in writing the manuscript.
